# Clinical, Environmental, and Serologic Surveillance Studies of Melioidosis in Gabon, 2012–2013

**DOI:** 10.3201/eid2101.140762

**Published:** 2015-01

**Authors:** W. Joost Wiersinga, Emma Birnie, Tassili A.F. Weehuizen, Abraham S. Alabi, Michaëla A.M. Huson, Robert A. G. Huis in ’t Veld, Harry K. Mabala, Gregoire K. Adzoda, Yannick Raczynski-Henk, Meral Esen, Bertrand Lell, Peter G. Kremsner, Caroline E. Visser, Vanaporn Wuthiekanun, Sharon J. Peacock, Arie van der Ende, Direk Limmathurotsakul, Martin P. Grobusch

**Affiliations:** University of Amsterdam, Amsterdam, the Netherlands (W.J. Wiersinga, E. Birnie, T.A.F. Weehuizen, M.A.M. Huson, R.A.G. in ’t Veld, C.E. Visser, A. van der Ende, M.P. Grobusch);; Albert Schweitzer Hospital, Lambaréné, Gabon (A.S. Alabi, H.K. Mabala, G.K. Adzoda, M. Esen, B. Lell, P.G. Kremsner, M.P. Grobusch);; Ex-Situ Silex Geoarchaeology, Leiden, the Netherlands (Y. Raczynski-Henk);; University of Tübingen, Tübingen, Germany (M. Esen, B. Lell, P.G. Kremsner, M.P. Grobusch);; Mahidol University, Bangkok, Thailand (V. Wuthiekanun, D. Limmathurotsakul);; University of Cambridge, Cambridge, UK (S.J. Peacock)

**Keywords:** Burkholderia pseudomallei, Burkholderia thailandensis, melioidosis, epidemiology, seroprevalance, Africa, Gabon, soil, sepsis, bacteria

## Abstract

*Burkholderia pseudomallei,* an environmental gram-negative bacillus, is the causative agent of melioidosis and a bio-threat agent. Reports of *B. pseudomallei* isolation from soil and animals in East and West Africa suggest that melioidosis might be more widely distributed than previously thought. Because it has been found in equatorial areas with tropical climates, we hypothesized that *B. pseudomallei* could exist in Gabon. During 2012–2013, we conducted a seroprevalance study in which we set up microbiology facilities at a large clinical referral center and prospectively screened all febrile patients by conducting blood cultures and testing for *B. pseudomallei* and related species; we also determined whether *B. pseudomallei* could be isolated from soil. We discovered a novel *B. pseudomallei* sequence type that caused lethal septic shock and identified *B. pseudomallei* and *B. thailandensis* in the environment. Our data suggest that melioidosis is emerging in Central Africa but is unrecognized because of the lack of diagnostic microbiology facilities.

## Abstract 2

*Burkholderia pseudomallei* and *B. thailandensis* are in the soil; a novel *B. pseudomallei* sequence type causes lethal septic shock.

The Tier 1 bio-threat agent *Burkholderia pseudomallei* is an environmental gram-negative bacillus and the cause of melioidosis, a disease characterized by sepsis, pneumonia, and abscess formation in almost any organ (*1*–*3*). *B. thailandensis* is closely related to *B. pseudomallei* but rarely causes disease in humans or animals; it is usually distinguished from *B. pseudomallei* by its ability to assimilate arabinose (*4*–*6*). Melioidosis mainly affects those who are in regular contact with soil and water and is associated with a mortality rate of up to 40% in resource-poor environments. The major regions to which melioidosis is endemic are Southeast Asia and tropical Australia (*1*,*2*). The northern tip of the Northern Territory in Australia and northeast Thailand represent hot spots, where annual incidence is up to 50 cases per 100,000 persons (*1*,*7*).

The emergence of melioidosis in Brazil is an example of increasing recognition of the disease in areas where it is probably endemic, and cases have become apparent as a result of enhanced awareness and diagnostics (*1*,*8*). Human *B. pseudomallei* infection has been reported from Malawi, Nigeria, The Gambia, Kenya, and Uganda; however, human cases in Africa seem to be few and isolated, although this finding could be the result of underrecognition and underreporting (*1*,*9*–*12*). Although reports of *B. pseudomallei* isolation from soil and animals in East and West Africa are limited, they suggest that melioidosis could be widely distributed across this region (*13*,*14*).

Given the equatorial tropical distribution of *B. pseudomallei* and *B. thailandensis*, we hypothesized that these bacteria are present in the central African country of Gabon, potentially causing disease. By conducting a seroprevalence study, an environmental survey, and setting up microbiology facilities for *B. pseudomallei* detection at a large referral hospital, we detected *B. pseudomallei* in soil and identified it as a cause of lethal infection in Gabon. We also detected *B. thailandensis* in environmental soil samples, indicating that this organism is also present in Gabon.

## Methods

### Study Sites and Populations

The study was performed in Moyen-Ogooué and Ngounié Provinces (combined population 162,000) in central Gabon; these 2 provinces cover an area of 56,285 km^2^ and consist of predominantly dense primary rain forest. For the seroprevalence surveillance study, 304 serum samples were collected from healthy nonfebrile school children (12–20 years of age) living in and around Lambaréné, the capital of Moyen-Ogooué Province; these children also participated in a chemoprophylaxis study for malaria (*15*). 

A prospective analysis of community-acquired bloodstream infections was performed at Albert Schweitzer Hospital (which admits ≈6,000 patients annually) in Lambaréné (population 24,000), in the Central African rain forest on the river Ogooué, Gabon. The rainy season starts in October and ends in June (including a short dry season in December–January). Mean annual rainfall is 1,981 mm (78 inches), which is equivalent to that in northeastern Thailand (*16*). Studies were approved by the Centre National de la Recherche Scientifique et Technologique, Libreville, and the scientific review committee of the Centre de Recherches Médicales de Lambaréné, Albert Schweitzer Hospital. 

### Prospective Analysis of Community-Acquired Bloodstream Infections

To obtain data about the prevalence and causes of community-acquired bloodstream infections in Lambaréné, we prospectively monitored all blood cultures for febrile patients admitted to Albert Schweitzer Hospital for 1 year (June 1, 2012–May 31, 2013) by using BacT/Alert PF (bioMérieux, Marcy l'Etoile, France). Criteria for ordering blood cultures were left to the discretion of the treating physician. Technicians and staff of the clinical microbiology laboratory received additional training on sample handling and processing (*17*,*18*). All oxidase-positive, gram-negative bacteria that were not *Pseudomonas aeruginosa* were further tested to determine whether they were *B. pseudomallei* by using the subculture and identification methods described below. Antimicrobial drug susceptibilities were determined by using Etest (bioMérieux) on Mueller-Hinton-agar (bioMérieux); when available, break points were defined as described (*19*).

### *B. pseudomallei* Antibody Detection by Indirect Hemagglutination Assay 

During May 2012, presence and titer of antibodies to *B. pseudomallei* in healthy schoolchildren were determined using by the indirect hemagglutination assay (IHA) as described (*20*,*21*), with pooled antigens prepared from 2 *B. pseudomallei* isolates from Thailand. An antibody titer of ≥1:40 was used as the cutoff value for seropositivity (*22*).

### Soil Sampling Study

**Figure 1:**
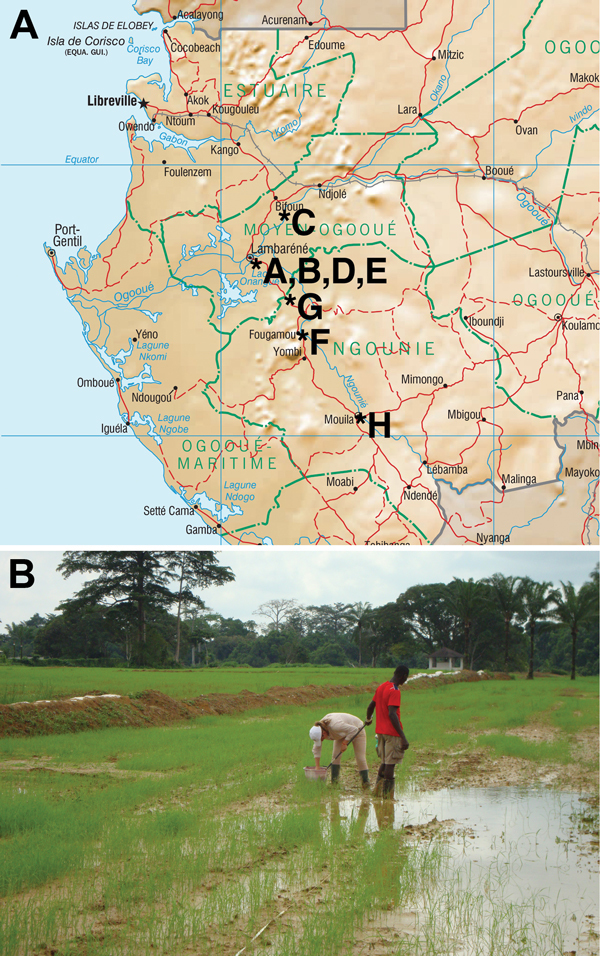
Environmental survey. A) Gabon, showing location of the 8 sites from which soil was sampled to test for the presence of *B. pseudomallei*, July 2012–September 2012. B) Soil sampling site no. H, a rice field near Mouila village.

During July 2012–September 2012, soil sampling to test for the presence of *B. pseudomallei* was based on consensus guidelines, and direct culture of soil in enrichment broth was performed (*17*,*23*). A total of 8 sites around the residences of children were selected on the basis of local maps and consultations with inhabitants throughout the provinces of Moyen-Ogooué (6 sites) and Ngounié (2 sites) and on known factors associated with the presence of *B. pseudomallei* (e.g., wet soil such as rice paddies or land use such as goat farming) (*17*) (Figure 1). Within each sampling area (50 × 50 m^2^), a fixed-interval sampling grid was used to collect 100 samples per field, 5 m apart. For each sample, 10 g of soil was collected from a depth of 30 cm, stored away from direct sunlight, and processed within 3 h. 

Isolation of potential *Burkholderia* spp. from soil was performed as described (*17*,*23*). In brief, 10 g of soil was diluted in 10 mL of threonine–basal salt solution plus colistin at 50 mg/liter (TBSS-C50 broth) containing crystal violet and was vortexed for 30 s before incubation at ≈42°C for 48 h. Ten μL of supernatant was subcultured onto Ashdown-agar and incubated and examined every 24 h for 7 days. *B. pseudomallei* was identified by colony morphology, positive oxidase test result, inability to assimilate arabinose, antimicrobial drug susceptibility pattern (*B. pseudomallei* is generally resistant to gentamicin and colistin but susceptible to amoxicillin/clavulanic acid [*1*,*2*]), and results of API 20NE (bioMérieux) and *B. pseudomallei*–specific (Bps) latex-agglutination tests (*18*,*24*,*25*). Positive results were confirmed with molecular analysis. Soil type was determined by standard lithologic and pedologic analysis of sediments; for this purpose, 2 extra samples were collected per site from a depth of 30 cm (*26*). Sediment properties were compared with properties of other (typical) samples from the same locations as described in the recently published Soil Atlas of Africa (*26*).

### Genetic and Phylogenetic Analyses

Genomic DNA was extracted by using a DNeasy Blood and Tissue Kit (QIAGEN, Valencia, CA, USA) to perform multilocus sequence typing (MLST) (*27*). Primers used to amplify fragments of the 7 housekeeping genes were identical to those described at the *Burkholderia* MLST website (http://bpseudomallei.mlst.net/misc/info2.asp). For isolate *B. thailandensis* D50, the primer narK-up was replaced by narK-upAMC 5′-TCTCTACTCGTGCGCTGGGG-3′. Sequences of the 7 gene fragments of isolates from Africa were concatenated and combined with those from a selection of 971 sequence types (STs) representing all *B. pseudomallei*, *B. mallei*, and *B. thailandensis* isolates in the *B. pseudomallei* MLST database. Concatenated sequences were aligned and analyzed by using MEGA-6 (http://www.megasoftware.net). A phylogenetic tree was constructed by using a neighbor-joining algorithm and the Kimura 2-parameter model. Bootstrap testing was performed for 500 repetitions. Whole-genome sequencing was performed by using the MiSeq platform (Illumina, San Diego, CA, USA) as described (*9*).

## Results

### Community-Acquired Bloodstream Infections

Of the 941 bacterial blood cultures, 77 (8.2%) were positive for bacteria. The most prevalent isolate was *Escherichia coli,* responsible for 8 (10.0%) bloodstream infections, followed by *Staphylococcus aureus* (6 [7.8%]) and *Salmonella enterica* (6 [7.8%], 5 of which were nontyphoidal salmonellae). Other organisms that were isolated at least 5 times included *Streptococcus pneumoniae* (5 [6.5%]), *Klebsiella pneumoniae* (5 [6.5%]), and *Enterobacter* spp. (5 [6.5%]). *B. pseudomallei* was isolated from 1 (1.4%) patient, described in the case report.

### Case Report

A 62-year-old Gabonese woman was hospitalized in January 2013 with a 7-day history of fever, cough, weakness, headache, vomiting, and a painful knee. She did not report coughing or shortness of breath. She had poorly controlled diabetes mellitus and was taking glibenclamide. She had no history of cardiopulmonary or renal disease, was receiving no long-term medications other than glibenclamide, and did not smoke. She was a retired school teacher but still engaged in family farming. Physical examination revealed blood pressure of 160/90 mm Hg, a pulse rate of 130 beats per minute, and a temperature of 40.5°C. She had a wound with an underlying abscess on her right leg, together with diffuse tenderness of the right knee with warmth, erythema, and limitation of active and passive ranges of motion because of pain and effusion. Neurologic, cardiovascular, and respiratory examinations revealed no abnormalities. Laboratory findings obtained at admission showed an elevated blood glucose level of 24 mmol/L but values within reference range for creatinine (0.85 mg/dL), leukocytes (9,800 × 10^3^ mm^3^), and hemoglobin (9.2 g/dL). No other blood or urine test was performed, and chest radiographs were not taken. On hospitalization day 1, treatment with amoxicillin/clavulanic acid was empirically initiated for sepsis. On day 2, the abscess was incised and drained, and on day 3 antimicrobial drug therapy was switched to ceftriaxone. Cultures of blood, wound, and synovial fluid grew identical gram-negative rods, which were initially classified as *Pseudomonas* spp. No other pathogens were detected. The patient’s clinical condition deteriorated, and she died of septic shock on day 8. A postmortem examination was not performed. 

After the patient’s death, the *Pseudomonas* species was classified as *B. pseudomallei* (patient strain Gb100) and confirmed by MLST and whole-genome sequencing. This isolate was later determined to be susceptible to trimethoprim/sulfamethoxazole, amoxicillin/clavulanic acid, ceftazidime, and meropenem **(**Table 1).

**Table 1:** Antimicrobial drug susceptibility of *Burkholderia pseudomallei* and *B. thailandensis* strains from Gabon, 2012–2013*

Drug	MIC, mg/L			
	Break point resistance	*B. pseudomallei* patient strain	*B. pseudomallei* soil strain C2	*B. thailandensis* soil strain D50
Amikacin	4†	96	96	128
Tobramycin	4†	16	24	24
Ciprofloxacin	1	0.75	1.0	0.5
Moxifloxacin	1‡	0.75	0.75	0.75
Meropenem	4	0.75	0.75	0.75
Ceftazidime	8	2	2	2
TMP/SMX	1/19	1	1	1
AMC	8/2	4	4	6
TZP	32/?§	1.5	1.5	3
Chloramphenicol	8	3	3	3
Tetracycline	4¶	1.5	2	8
Polymyxin B	NA#	>1,024	>1,024	>1,024

*Bacterial isolates were tested for their susceptibility to antimicrobial agents. MIC (MICs; mg/L) were determined by E-test on Mueller-Hinton-agar. When available break points were defined as described [19]. AMC, amoxicillin/clavulanic acid; NA, not applicable; TMP/SMX, trimethoprim/sulfamethoxazole, TZP, piperacillin/ tazobactam. †Break point for gentamicin was used. ‡Break point for ciprofloxacin was used. §Break point available for piperacillin only. ¶Break point for doxycycline was used.  #Intrinsic resistance.

### Seroprevalence 

Of the 304 healthy schoolchildren for whom serum samples were tested for *B. pseudomallei* antibodies, 143 (47.0%) were male. Details for this cohort have been reported previously (*15*). For 43 (14.1%) children, an IHA titer was detectable; titers ranged from 1:10 to 1:80 (median 1:10, interquartile range 1:10–1:20). For 5 (1.6%) children, IHA titer was >1:40, which has been used as the cutoff value for seropositivity (*22*). None of the children had an IHA titer >1:160, which is considered by several centers in Thailand to support a diagnosis of melioidosis in patients with clinical features consistent with this diagnosis.

### Environmental Isolates

The predominant soil type in this area of Gabon was ferralsol, which is red and yellow weathered soil. The only exception was samples taken from a rice paddy near Mouila village, where the soil was gleysol (clay, a hydric soil saturated with groundwater long enough to develop a characteristic gleyic color pattern) (Table 2). *B. pseudomallei* was isolated from 21 (3%) of 800 soil samples taken from 3 (38%) of the 8 sample sites; the maximum number of positive samples for 1 site was 14 (14%) (Table 2). The biochemical profiles of all isolates were in accordance with *B. pseudomallei* (API 20NE code 1156576). The antibiogram of *B. pseudomallei* soil strain C2 is shown in Table 1. 

**Table 2:** Geographic features and distribution of *Burkholderia pseudomallei* strains at 8 sampling sites in Moyen-Ogooué and Ngounié Provinces, Gabon, 2012–2013*

Site	Nearest village	Elevation, m	Land use	Soil type	Soil description	Sample holes positive, %
A	Lambaréné, Albert Schweitzer Hospital; lat. S 00°40′40.5, long. E 010°13′49.7	34	Football (soccer) field	Ferralsol	Yellowish-brown, clay fluvial sediments, not strongly humic, some gravel, poorly sorted sediment, decalcified	14
B	Lambaréné, Adouma; lat. S 00°40′50.2, long. E 010°13′31.5	14	Riverbed that is dry most of the year	Ferralsol, clay, orange, dry	Brownish yellow, clay fluvial sediments, moderately humic, some gravel, strong indicators of human interference	0
C	Makouké; lat. S 00°28′30.8, long. E 010°24′34.7	20	Cattle ranch	Ferrasol, orange, little stones, hard, rocky, less hard, orange	Yellowish brown, clay fluvial sediments, not strongly humic, some gravel, poorly sorted sediment, decalcified	4
D	Lambaréné, Adiwa; lat. S 00°41′06.0, long. E 010°13′43.5	8	Next to school (with Bps IHA positivity)	Ferralsol	Brownish yellow, clay fluvial sediments, moderately humic, some gravel, strong indicators of human interference	3
E	Lambaréné, Petit Paris 3; lat. S010°42′40.4, long. E 010°15′20.7	35	Cattle ranch	Savannah/ferralsol	Yellowish gray, well-sorted clay, weakly humic	0
F	Fougamou; lat. S 01°18′40.3, long. E 010°37′14.4	88	Savannah, grassland	Savannah/ferralsol	Yellowish gray, well-sorted clay, weakly humic	0
G	Massika II; lat. S 00°40′40.7, long. E 010°13′51.4	55	Football pitch	Ferralsol	Reddish brown, clay fluvial sediments, not strongly humic, sediment, decalcified	0
H	Mouila; lat. S 01°51′27.8, long. E 011°02′37.7	92	Rice paddy	Gleysol	Greyish yellow clay with ferric concretions, gleyic features, probably associated with rice cultivation	0

*lat., latitude; long., longitude.

**Figure 2:**
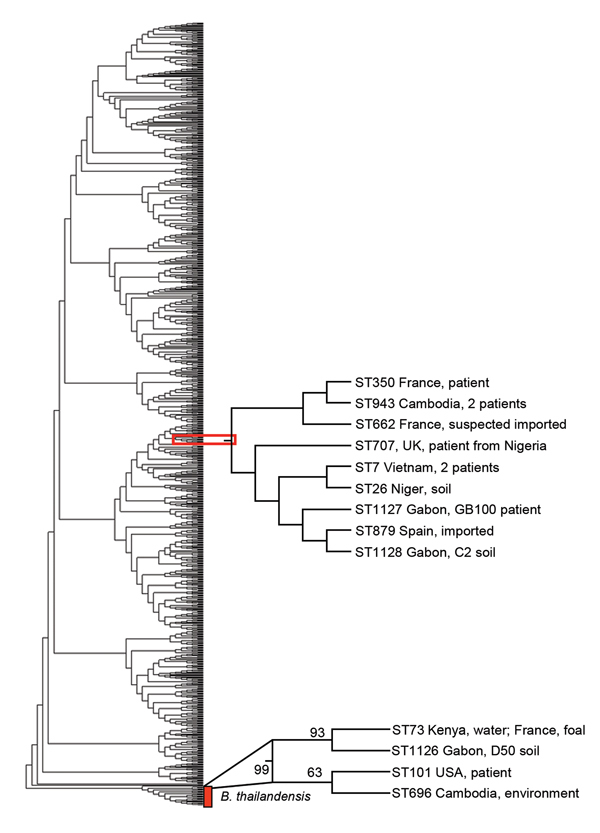
Phylogenetic tree of *Burkholderia pseudomallei* and *B. thailandensis* strains from Gabon, 2012–2013. Phylogenetic analysis by multilocus sequence typing amplification (MLST) of isolate Gb100 (from 62-year-old patient who died of melioidosis), *B. pseudomallei* soil isolate C2 (sample collected at site C), and *B. thailandensis* soil isolate D50 (sample collected at site D), together with sequence types representing all *B. pseudomallei* and *B. thailandensis* isolate accessible in the MLST database. Phylogenetic tree was constructed by using the neighbor-joining algorithm with the Kimura 2-parameter model. Bootstrap test was for 500 repetitions. Sequence type labels were omitted for simplicity. Position of the isolates from Gabon, including their closest relatives, are indicated.

The closely related *B. thailandensis* coexists with *B. pseudomallei* in the soil in Southeast Asia and Australia and is generally considered avirulent (*5*,*28*). We also identified *B. thailandensis* in the soil of Gabon (Figure 2). This strain, termed *B. thailandensis* soil strain D50, was positive by Bps latex agglutination. This *B. thailandensis* strain, API 20NE code 1157577, was susceptible to trimethoprim/sulfamethoxazole, amoxicillin/clavulanic acid, ceftazidime, and meropenem (Table 1).

### Genetics and Phylogeny *of Burkholderia* spp. Strains 

The 3 isolates from Gabon contained previously described MLST alleles but belonged to novel STs. The patient isolate Gb100 (ST1127) and soil isolate C2 (ST1128) were single-locus variants and differed by 1 nt in the *narK* sequence only. Patient isolate Gb100 was also a single-locus variant of ST707 (single-nucleotide substitution in *ndh*). The only *B. pseudomallei* strain with ST707 in the database had been isolated in 2010 from a patient in the United Kingdom, 6 weeks after the patient had returned from a trip to Nigeria (*12*). The soil isolate C2 (ST1128) was a single-locus variant of ST7 (single-nucleotide substitution in *ndh*) and ST879 (single-nucleotide substitution in *lipA*). ST7 was represented by 2 isolates in the MSLT database, both isolated in 1963 from patients in Vietnam. The *B. pseudomallei* ST879 strain was isolated in 2011 from a patient in Spain, who had returned from a trip through Madagascar and 14 countries in West Africa (*11*). The soil isolate D50 (ST1126) was a single-locus variant of ST73. This ST is represented in the database by 2 *B. thailandensis* strains, 1 isolated from a foal in France and 1 isolated from the environment in Kenya. Phylogenetic analysis of the Gabon isolates together with 971 STs obtained from the MLST database by using the aligned concatenated sequences of the 7 loci in the neighbor-joining algorithm with the Kimura 2-parameter model showed that the patient isolate Gb100 and soil isolate C2, found near the community of the patient, grouped together with 7 STs. These 7 STs represented 10 *B. pseudomallei* strains isolated in Cambodia (2 strains), Vietnam (2 strains), Niger, Nigeria, Spain (imported), France (2 strains [1 imported]), and the United Kingdom (imported) (Figure 2). Again, patient isolate Gb100 and soil isolate C2 are most closely related to ST879. Soil isolate D50 grouped together with 3 STs representing 4 *B. thailandensis* strains isolated from Kenya, France, the United States, and Cambodia. Using this approach, we showed that the closest relatives of the strain that infected and eventually killed the patient reported here were ST879 and the strain isolated from soil around her community. Our whole-genome sequencing sample data have been submitted to a project that is undertaking whole-genome sequencing on a large number of *B. pseudomallei* isolates from around the world. This approach is anticipated to offer superior resolution of the global phylogeny of *B. pseudomallei* (*9*).

## Discussion

We detected a case of melioidosis in a human in central Africa, confirmed the presence of *B. pseudomallei* in the environment in Gabon, and isolated *B. thailandensis* from an environmental sample from that part of the world. The low rate of antibody seropositivity among healthy children combined with the low prevalence of *B. pseudomallei* cultured from blood of patients in a local hospital, however, suggest that melioidosis is rare in this setting.

Only 4 of the 13 melioidosis cases acquired by humans in Africa and reported in the literature have been PCR confirmed (*9*–*12*,*29*–*34*). We show with phylogenetic analysis that the newly identified patient isolate Gb100 groups with a *B. pseudomallei* isolate from a patient from Spain who had traveled across West Africa and Madagascar (*12*). *B. pseudomallei* seropositivity was reported during a World Health Organization investigation into an outbreak of severe pneumonia in the northeastern of the Democratic Republic of Congo (Eric Bertherat, pers. comm.) (*35*). However, in that study some of the *B. pseudomallei*–seropositive cases diagnosed as melioidosis were later diagnosed as plague, calling into question the value of serology-based testing in this setting (*35*). The predominant soil type at the sites from which *B. pseudomallei* was isolated was similar to the soil type from which *B. pseudomallei* strains were isolated in Cambodia (*26*,*36*). The low rate of *B. pseudomallei* positivity per site points toward a relatively low abundance of *B. pseudomallei* in Gabon soil when compared with highly melioidosis-endemic areas in Southeast Asia and Australia (*21*,*37*). The true distribution of melioidosis in Africa remains uncertain, but we now can expand this area toward the central African country of Gabon.

The genus *Burkholderia* comprises >30 species, of which *B. pseudomallei* and *B. mallei* are considered the most pathogenic (*2*,*38*). The isolation of *B. thailandenis* from soil in Gabon extends our knowledge of the geographic distribution of this species. This strain was positive by Bps latex agglutination; this finding is in agreement with previous findings of a *B. thailandensis* strain from Thailand with a Bps-like capsular polysaccharide variant that also had a positive Bps latex-agglutination result (*39*). Our phylogenic analysis shows a divergence between the strain from Gabon and the original *B. thailandenis* E264 from Thailand, which is the most studied strain (*4*,*5*). Evidence of the presence of this bacterium in Africa will have implications for bacterial identification in clinical laboratories, diagnostic serology assays, and environmental studies.

Our study has several limitations. *B. pseudomallei* serology can be misleading; false- positive results are a major concern (*40*). Clearly, for assessing exposure to *B. pseudomallei*, an accurate, inexpensive, simple serologic assay is needed. In the interim, however, serologic evidence of exposure should be based on assays with known sensitivity and specificity against culture-confirmed melioidosis, and, to our understanding, the IHA is the best test for identifying melioidosis cases. Given the nature of working in a resource-poor environment, only limited information is available on the patient reported here (e.g., no imaging was performed to investigate the presence of deeper abscesses). With regard to the environmental study, *B. pseudomallei* is known for its capacity to survive in water and has been reported to be present in the air during severe weather (*17*); we, however did not investigate its presence in water and air in Gabon in this study. Furthermore, we cannot dismiss the possibility of error during soil sampling although guidelines for environmental sampling of *B. pseudomallei* were followed (*17*).

In summary, we identified *B. pseudomallei* and *B. thailandensis* in the Gabon environment and discovered a novel *B. pseudomallei* ST that can cause lethal septic shock. *B. pseudomallei* is probably an underrecognized cause of disease in central Africa. We propose that melioidosis occurs in central Africa but that it is unrecognized because of the lack of diagnostic microbiology facilities.
